# Time-Dependent Differences in the Effects of Oleic Acid and Oleyl Alcohol on the Human Skin Barrier

**DOI:** 10.1021/acs.molpharmaceut.3c00648

**Published:** 2023-11-11

**Authors:** Andrej Kováčik, Monika Kopečná, Iva Hrdinová, Lukáš Opálka, Mila Boncheva Bettex, Kateřina Vávrová

**Affiliations:** † Skin Barrier Research Group, Charles University, Faculty of Pharmacy in Hradec Králové, Akademika Heyrovského 1203, 50005 Hradec Králové, Czech Republic; ‡ HALEON CH SARL, Route de l’Etraz 2, Case Postale 1279, Nyon 1260, Switzerland

**Keywords:** topical drug delivery, permeation enhancer, penetration enhancer, skin barrier, lipid interactions, infrared spectroscopy, diclofenac

## Abstract

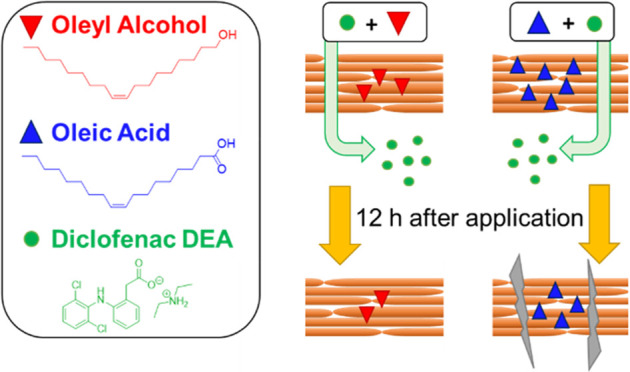

Oleic acid and oleyl alcohol are commonly used permeation and penetration enhancers to facilitate topical drug delivery. Here, we aimed to better understand the mechanism of their enhancing effects in terms of their interactions with the human skin barrier using diclofenac diethylamine (DIC-DEA), a nonsteroidal anti-inflammatory drug for topical pain management. Oleic acid promoted DIC-DEA permeation through ex vivo human skin more rapidly than oleyl alcohol (both applied at 0.75%) due to fluidization of stratum corneum lipids as revealed by infrared spectroscopy. After 12 h, the effect of these enhancers on DIC-DEA permeation leveled off, fluidization was no longer evident, and skin permeabilization was mainly due to the formation of fluid enhancer-rich domains. Contrary to oleyl alcohol, oleic acid adversely affected two indicators of the skin barrier integrity, transepidermal water loss and skin electrical impedance. The content of oleyl alcohol in the stratum corneum was lower than that of oleic acid (even 12 h after the enhancers were removed from the skin surface), but it caused higher DIC-DEA retention in both epidermis and dermis compared to oleic acid. The effects of oleyl alcohol and oleic acid on DIC-DEA permeation and retention in the skin were similar after a single and repeated application (4 doses every 12 h). Thus, oleyl alcohol offers several advantages over oleic acid for topical drug delivery.

## 1 Introduction

Topical drug delivery is a method of administering drugs through the skin to achieve local therapeutic effects, with several advantages over systemic drug delivery, such as reduced systemic side effects and improved patient compliance.^1^ For example, topical administration of nonsteroidal anti-inflammatory drugs such as diclofenac (DIC) is a clinically successful method to target pain directly at its site of origin.^2^ The efficacy of such topical products is critically determined by the drug form itself (e.g., various salts^3^) as well as by the formulation ingredients.^4−6^

**Figure 1:**
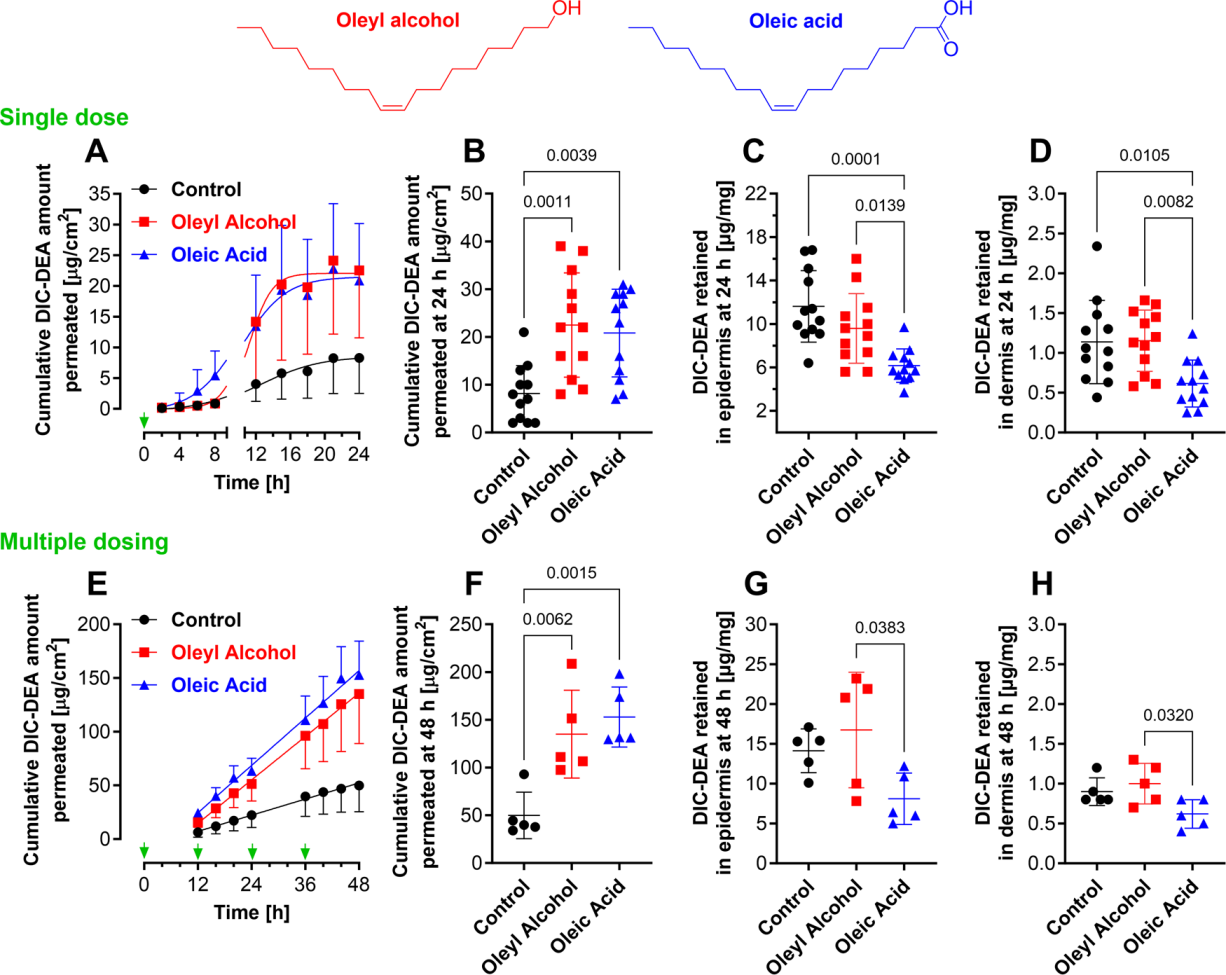
Chemical structure of the studied permeation enhancers and their impact on the skin permeation and retention of diclofenac diethylamine (DIC-DEA) when applied as a single dose (A–D) or 4 doses every 12 h (E–H), in the absence of enhancer (shown in black), together with oleyl alcohol (shown in red) and oleic acid (shown in blue). Cumulative permeation profiles of DIC-DEA (A, E); comparison of the cumulative 24 or 48 h DIC-DEA permeation (B, F), retention in the epidermis (C, G) and the dermis (D, H). Data are presented as mean ± SD and individual data points. *n* = 12 (three skin donors, four replicates per donor) in the single-dose experiment (A–D) or *n* = 5 in the multiple-dose setup (E, F). The *p* values lower than 0.05 are indicated.

The major barrier to effective topical (and transdermal) drug delivery is the outermost skin layer, the stratum corneum (SC), particularly its highly hydrophobic extracellular lipid matrix. Therefore, techniques used to improve topical or transdermal drug delivery, such as chemical permeation enhancers (also termed penetration enhancers, accelerants, absorption promoters), target these highly ordered and densely packed lipids (ideally reversibly).^7−9^ Oleic acid and oleyl alcohol are two enhancers that have been extensively studied and are commercially used for topical drug delivery.^10−20^ Oleic acid is a monounsaturated ω-9 fatty acid found naturally in many vegetable oils, such as olive oil, and oleyl alcohol is a fatty alcohol derived from oleic acid (Figure 1). Both enhancers have the same 18-carbon chain with a *cis-*double bond in the middle that causes the characteristic chain kink that prevents effective chain packing and makes them liquid at room temperature (melting points of 13 °C for oleic acid and 5 °C for oleyl alcohol). Both are also highly lipophilic (log *P* > 7) and have a high affinity for SC lipids. They differ in their polar head structure, a carboxyl function in oleic acid, and a hydroxyl function in oleyl alcohol. The carboxyl is bulkier compared to hydroxyl, forms more hydrogen bonds, and can be ionized to negatively charged carboxylate at physiologically relevant pH values. Oleic acid has been reported to either disorder the SC lipids or form discrete domains that facilitate drug permeation or both.^10−16^ More fluid chains have also been reported in oleyl alcohol-treated skin.^21^ Skin irritation has been reported for 4.5–10% oleic acid,^16,22,23^ whereas up to 10% oleyl alcohol induced no discernible change in the histologic appearance of nude mouse skin.^23^

Commercially available over-the-counter DIC products containing oleyl alcohol or oleic acid have demonstrated equivalent 24 h transdermal delivery of DIC in vitro,^24^ but it is unclear whether these two enhancers act in the same way in terms of their permeation and retention in the SC, time-dependent interactions with skin barrier components, effect on water loss, and the rate of recovery from the skin barrier disruption after removal of the product. These are all factors that are important to consider when formulating topical products intended to be well tolerated even upon frequent or extended use. In addition to the drug amount that permeates through the skin, it is also important to understand the effect of these permeation enhancers on the drug retention in the skin (creating reservoir)—a factor important for the sustained release and action of topical medicinal products.^25−27^

Here, we aimed to better understand the differences in the permeation-enhancing effects of oleic acid and oleyl alcohol in relation to their interactions with the human skin barrier. We compared the effects of oleyl alcohol and oleic acid at a concentration found in commercially available topical gels (0.75% w/v) on the permeation and retention of 2.32% (w/v) DIC diethylamine (DIC-DEA; equivalent to 2% free DIC) in ex vivo human skin. We also investigated the effect of the enhancers on two indicators of the skin barrier function, the transepidermal water loss (TEWL) and electrical impedance, and the reversibility of these parameters over time after removal of the enhancers from the skin surface. In addition, the interactions of oleic acid and oleyl alcohol with the skin barrier over time were investigated by Fourier transform infrared (FTIR) spectroscopy on human skin treated with deuterated enhancers (to separate absorption peaks arising from the endogenous SC lipids and the enhancers). For this purpose, deuterated oleyl alcohol-*d*_33_ was synthesized.

## 2 Experimental Section

### 2.1 Chemicals

Chemicals were purchased from Sigma-Aldrich (Schnelldorf, Germany) and used as received. TLC was performed on Merck aluminum plates with silica gel 60 F_254_. Merck Kieselgel 60 (0.040–0.063 mm) was used for column chromatography. Water was purified through a Millipore Q purification system.

### 2.2 Synthesis of Deuterated Oleyl Alcohol-*d*_33._

Oleic acid-*d*_34_ (23 mg; 0.073 mmol) was dried in high vacuum, dissolved in 1 mL of dry THF, and cooled in an ice bath to 0 °C. 220 μL (0.22 mmol) of 1 M LiAlH_4_ solution in THF was added dropwise, and the reaction mixture was stirred for an additional 1 h at 0 °C and 30 min at room temperature. The reaction was quenched by a slow addition of 3 mL of water and 1 mL of 1 M HCl. The reaction mixture was extracted with diethyl ether (4 × 10 mL), and the organic phase was dried with sodium sulfate and evaporated to dryness. The crude product was purified using column chromatography on silica with a mobile phase hexane/ethyl acetate 10:1 (v/v) to yield 18 mg (82%) of the product as a white semisolid. Rf (hexane/ethyl acetate; 4:1, v/v) = 0.4. FTIR (ATR): ν_max_ = 2198, 2096, 1088, 1050 cm^–1^.

### 2.3 Human Skin

Human skin was obtained from seven Caucasian females (28–59 years) who had undergone abdominoplasty with their written informed consent. The study was approved by the Sanus Surgical Centre Ethics Committee (3/11/2022) according to the principles of the Declaration of Helsinki. Subcutaneous fat was carefully removed from the tissue, and the remaining full-thickness skin fragments were washed with water and saline, dabbed dry, and stored at −20 °C. Prior to the permeation experiment, the frozen human skin was slowly thawed and cut to a thickness of approximately 400 μm using an Acculan 3TI dermatome (Aesculap).

### 2.4 DIC-DEA Samples for Permeation Experiments

The DIC-DEA samples for permeation experiments contained DIC-DEA (2.32%) in a mixture of isopropyl alcohol/propylene glycol/water (20:6:74, v/v/v) with or without oleyl alcohol or oleic acid at 0.75% (w/v). All samples were thoroughly mixed and incubated at 32 °C for 24 h. The DIC-DEA solubility in this solvent mixture is ∼100 mg/mL and is slightly increased in the presence of oleyl alcohol and oleic acid (118 and 129 mg/mL) with no significant difference between the enhancers. Thus, the 2.32% DIC-DEA concentration corresponds to 23, 20, and 18% saturation in the control, oleyl alcohol, and oleic acid-containing samples, respectively. Therefore, the thermodynamic activities of the drug in the studied formulations are similar.

### 2.5 Skin Permeation Experiments

The permeation-enhancing potencies of the enhancers were evaluated on human skin in Franz diffusion cells. Dermatomed human skin was cut into 2 cm × 2 cm pieces, fixed in Teflon holders with 1 cm^2^ circular permeation areas, and mounted in Franz cells with the epidermal side up. The acceptor compartment (7.0 ± 0.5 mL) was filled with phosphate-buffered saline (PBS) at pH 7.4, containing 0.005% gentamicin. The cells were visually inspected for leaks and entrapped air bubbles (before and during the experiment) and placed on a magnetic stirrer in a water bath at 32 ± 1.0 °C. Skin integrity was evaluated by using electrical impedance. The assembled Franz cells were equilibrated for 30 min, and then 500 μL of PBS was applied to the SC surface of the skin. After 1 h of equilibration, the electrical impedance of the skin samples was assessed (see below). All skin fragments met the predefined cutoff of impedance ≥10 kΩ × cm^2^. After the impedance measurement, PBS was carefully removed from the SC surface of the skin, and the tissue was dried with cotton swabs.

Next, each of the DIC-DEA samples (10 μL/cm^2^) was applied on the surface of 12 skin fragments (3 skin donors, 4 replicates each) without occlusion to mimic real-life application as closely as possible. For technical convenience, a 300 μL aliquot of the acceptor fluid was collected at 2, 4, 6, and 8 h postdosing in one experiment, and at 12, 15, 18, 21, and 24 h in another one. The concentration of DIC-DEA was measured by high-performance liquid chromatography (HPLC). The cumulative amount of DIC-DEA permeated through the skin was corrected for the acceptor phase replacement and acceptor volume and plotted against time. After the permeation experiment, the cells were dismounted, the skin was carefully washed with PBS, and the permeation area of the skin (1 cm^2^) was punched out. After heating the skin fragments to 80 °C for 1 min, the epidermis was peeled off the dermis, individually weighed, and extracted by 1 mL of an extraction solvent (identical to the mobile phase for the HPLC analysis, see below) for 24 h.^28^ The skin was heated in aluminum foil (not in an aqueous medium) to prevent drug extraction during epidermal isolation. Although we cannot exclude minor drug redistribution between the epidermis and dermis during this procedure (1 min), we believe it does not affect the outcome of the experiment, as the same trends were found in the epidermis and dermis.

### 2.6 High-Performance Liquid Chromatography (HPLC)

DIC-DEA was determined by isocratic reversed-phase HPLC using a Shimadzu Prominence instrument (Shimadzu, Japan) on a LiChroCART 250-4 (LiChrospher 100 RP-18, 5 μm) column at 30 °C using a mixture of acetonitrile/water/acetic acid (90:60:5, v/v/v) at 2.0 mL/min. The injection volume was 20 μL; the effluent was measured at 275 nm, and the retention time was 3.6 min. The calibration was linear in the range of 0.5–60 μg/mL.

### 2.7 Reversibility of Enhancer Effects on Transepidermal Water Loss (TEWL) and Electrical Impedance

Human skin was mounted in Franz diffusion cells in the same way as that for the permeation studies. After 1 h of equilibration, basal TEWL and impedance values (lower impedance values imply less opposition to the passage of alternating electric current) were recorded. Then, 0.75% oleyl alcohol or oleic acid in a mixture of isopropyl alcohol/propylene glycol/water (20:6:74, v/v/v), and a mixture of isopropyl alcohol, propylene glycol, and water as control were applied on the skin (150 μL/cm^2^). After 24 h, the remaining DIC-DEA sample was washed with PBS and the skin surface was gently blotted dry. At 1, 4, 8, 12, and 24 h after sample removal, TEWL and impedance values were recorded. TEWL was measured with an AquaFlux AF 200 (Biox Systems Ltd., London, UK), using the condenser-chamber measurement method at 27 ± 1.0 °C and 44 ± 3% relative air humidity. Electrical impedance was measured by using LCR meter 4080 (Conrad Electronic, Hirschau, Germany) operated in parallel mode with an alternating frequency of 120 Hz.^29,30^

### 2.8 Fourier Transform Infrared (FTIR) Spectroscopy

FTIR spectra were collected at ambient temperature from dermatomed skin treated with samples containing or not deuterated enhancers (0.75% in the same solvent mixture as above) in Franz diffusion cells (same conditions as for the permeation experiment) using a Nicolet 6700 FTIR spectrometer (Thermo Scientific, Waltham) equipped with a single-reflection MIRacle attenuated total reflection ZnSe crystal. First, the enhancer or solvent samples were applied to the skin at the dose used in the permeation experiments (10 μL/cm^2^) for 8 or 24 h or at the dose used in the reversibility experiments (150 μL/cm^2^) for 24 h. Next, FTIR spectra were collected at 1, 4, 8, and 12 h after the removal of the enhancer or solvent sample. The spectra were generated by the coaddition of 128 scans recorded at a 2 cm^–1^ resolution.^31^ Peak positions and intensities were determined after rubber band baseline correction without smoothing. For the relative quantification of the selected components, we used peak intensities after baseline correction.

### 2.9 Data Analysis

The statistical analysis ANOVA with Dunnett’s post-test was performed with GraphPad Prism 6.07 (GraphPad Software). In view of the relatively low number of donors sufficient for this proof-of-concept study, data are reported as mean ± standard deviation (SD).

## 3 Results and Discussion

### 3.1 Oleyl Alcohol and Oleic Acid at 0.75% Have Comparable Effects on the 24 h DIC-DEA Permeation after One Dose or on the 48 h DIC-DEA Permeation after Four Doses, but Oleic Acid Causes Faster Permeation in the First 8 h

The permeation profiles of samples containing the two enhancers differed considerably during the first 2–8 h (Figure 1A). Oleic acid caused faster DIC-DEA permeation compared to both oleyl alcohol and control, and a higher DIC-DEA cumulative amount was found in the acceptor at 8 h with oleic acid (5.4 ± 4.1 μg/cm^2^) compared to oleyl alcohol (0.85 ± 1.2 μg/cm^2^) and control (0.85 ± 0.66 μg/cm^2^). This initial difference, however, leveled off with time. Figure 1A,B shows comparable effects of oleyl alcohol and oleic acid on the amount of DIC-DEA permeating through ex vivo human skin into the acceptor compartment over 24 h. Oleyl alcohol and oleic acid increased the cumulative amount of DIC-DEA in the acceptor by 2.8-fold (23 ± 3.1 μg/cm^2^) and 2.6-fold (21 ± 9.2 μg/cm^2^), respectively, compared to the control (solvent alone; 8.2 ± 5.8 μg/cm^2^). The amount of DIC-DEA found in the acceptor after 24 h corresponds to 3.5, 9.9, and 9.1% of the applied dose (232 μg/cm^2^ DIC-DEA) in the control sample and those with oleic alcohol and oleic acid, respectively.

Because topical DIC-DEA is applied repeatedly, typically twice daily, we verified DIC-DEA permeation after repeated dosing. Given the limitations of the in vitro experiment, we applied 4 doses 12 h apart and monitored DIC-DEA in the acceptor for 48 h (Figure 1E–H). In contrast to a single dose, repeated application every 12 h resulted in an almost linear increase in the cumulative amount of the drug in the acceptor solution. Oleyl alcohol and oleic acid increased the cumulative amount of DIC-DEA in the acceptor by 2.7-fold (135 ± 46 μg/cm^2^) and 3.1-fold (153 ± 31 μg/cm^2^), respectively, compared to the control (solvent alone; 50 ± 24 μg/cm^2^). The amount of DIC-DEA found in the acceptor after 24 h corresponds to 5.4, 14.5, and 16.5% of the applied dose (4 × 232 μg/cm^2^ DIC-DEA) in the control sample and those with oleic alcohol and oleic acid, respectively. Thus, the effect of the investigated enhancers on DIC-DEA permeation is very similar after one and four applications on the skin, with no differences between them.

**Figure 2:**
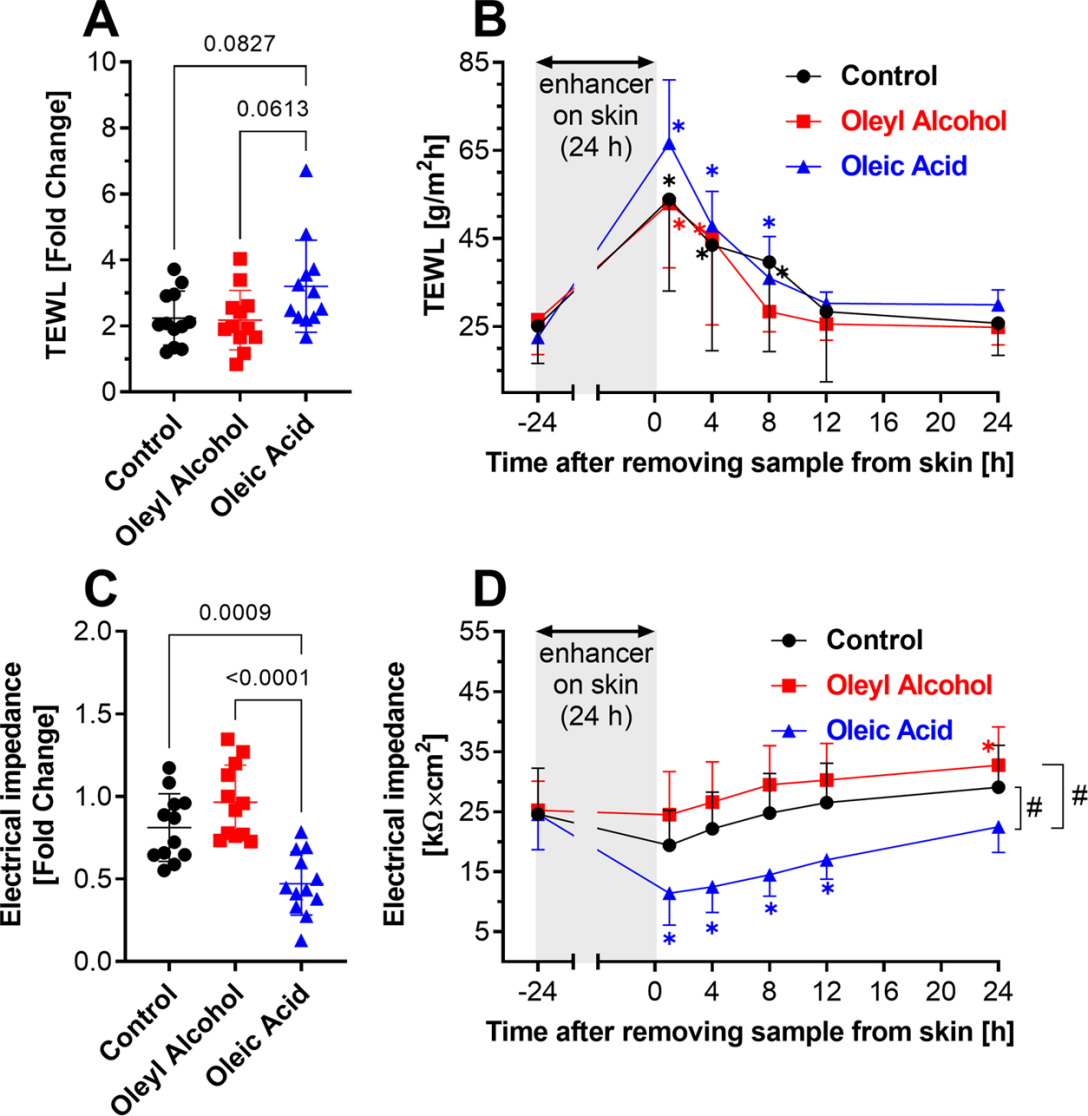
Effects of enhancers on the TEWL and electrical impedance of the skin. TEWL (A) and electrical impedance (C) fold change induced by 24 h application of the studied samples without enhancers (control, i.e., isopropyl alcohol, propylene glycol, and water at 20:6:74, v/v/v–shown in black) or with 0.75% oleyl alcohol (shown in red) or oleic acid (blue). The exact *p* values for the relevant comparisons are shown. Evolution of TEWL (B) and electrical impedance (D) values over time after removal of the samples from the skin surface. Data are presented as mean ± SD, *n* = 12 (three skin donors, four replicates per donor). Asterisks indicate significant differences vs baseline (i.e., before application, at time −24 h), and hashes indicate differences between samples as indicated, at *p* < 0.05.

### 3.2 Oleyl Alcohol Causes Higher Retention of DIC-DEA in the Skin Compared to Oleic Acid after Both Single and Multiple Doses

The amount of DIC-DEA retained in the skin after 24 h was higher when co-applied with oleyl alcohol than when co-applied with oleic acid in both the epidermis (9.58 ± 3.21 μg/mg with oleyl alcohol and 6.16 ± 1.54 μg/mg with oleic acid, Figure 1C) and the dermis (1.15 ± 0.38 μg/mg with oleyl alcohol and 0.62 ± 0.29 μg/mg with oleic acid, Figure 1D). Thus, 61, 53, and 30% of the DIC-DEA dose applied to the skin was found in the skin (epidermis + dermis) after application of the control, oleyl alcohol, and oleic acid samples, respectively. Notably, oleic acid lowered the DIC-DEA amount retained in the skin compared with the control solvent mixture.

Similar results were found after four doses. The amount of DIC-DEA retained in the skin after 48 h was higher when co-applied with oleyl alcohol than when co-applied with oleic acid in both the epidermis (16.7 ± 7.2 μg/mg with oleyl alcohol and 8.1 ± 3.2 μg/mg with oleic acid, Figure 1G) and the dermis (1.00 ± 0.26 μg/mg with oleyl alcohol and 0.62 ± 0.18 μg/mg with oleic acid, Figure 1H). Thus, 14, 17, and 8% of the DIC-DEA dose applied to the skin was found in the skin (epidermis + dermis) after application of the control, oleyl alcohol, and oleic acid samples, respectively, after four doses. Thus, repeated application increases the amount of DIC-DEA reaching the acceptor but does not significantly increase the amount retained in the skin.

The higher DIC-DEA skin retention caused by oleyl alcohol compared to oleic acid may potentially be advantageous, as the drug reservoir formed by topical nonsteroidal anti-inflammatory drug formulations in the skin has been shown to be important for the sustained, long-lasting release of the drugs into underlying tissues.^4,32−34^ Of course, translation to an in vivo situation must be done with caution as clearance will always be an issue in vitro due to the lack of microcirculation. In this respect, our repeated application setup provided quite interesting data, as the drug concentration in the skin remained about the same compared with a single dose, and only the drug concentration in the acceptor phase increased. Thus, the relatively large volume of a stirred acceptor that adequately dissolves the drug is likely to ensure (at least partially) clearance from the skin.

### 3.3 Contrary to Oleic Acid, the Permeation-Enhancing Action of Oleyl Alcohol Does Not Impact Adversely TEWL or the Skin Electrical Impedance

Next, we compared the effect of oleyl alcohol and oleic acid at 0.75% relative to solvent alone on two indicators of the skin barrier integrity, transepidermal water loss (TEWL; Figure 2A) and skin electrical impedance (Figure 2C), as well as the reversibility of these effects (Figure 2B,D). When a dose of 10 μL/cm^2^ was applied to the skin, these two methods were not sensitive enough to detect any changes (fold change close to 1; data not shown). To detect any differences, we applied a higher sample volume than that in the permeation experiments (150 μL/cm^2^) for 24 h. We are aware that this dose is excessive, but we believe it is appropriate if we want to determine the potential risk or differentiate the effect of the two formulations. Then, we removed the samples (using a previously validated procedure^28^) and monitored TEWL or impedance for another 24 h. The 24 h application of oleic acid caused a higher increase of TEWL compared to the application of oleyl alcohol or solvent alone (Figure 2A). After removal of all three topical samples from the skin, their effects were reversible; i.e., TEWL decreased to values comparable to those before application (Figure 2B). Oleic acid had a significantly higher effect on the electrical impedance compared to oleyl alcohol and the control (Figure 2C), which was not completely reversed even 24 h after removal of the samples from the skin (Figure 2D). Thus, the oleic acid disruption of the skin barrier persisted (at least partially) for several hours after discontinuation of the treatment.

### 3.4 After 8 h Application, Oleic Acid but Not Oleyl Alcohol Fluidizes the Skin Barrier Lipids, Whereas after 24 h, the Primary Mechanism of Action of Both Enhancers Appears to Be the Formation of Fluid Domains

**Figure 3:**
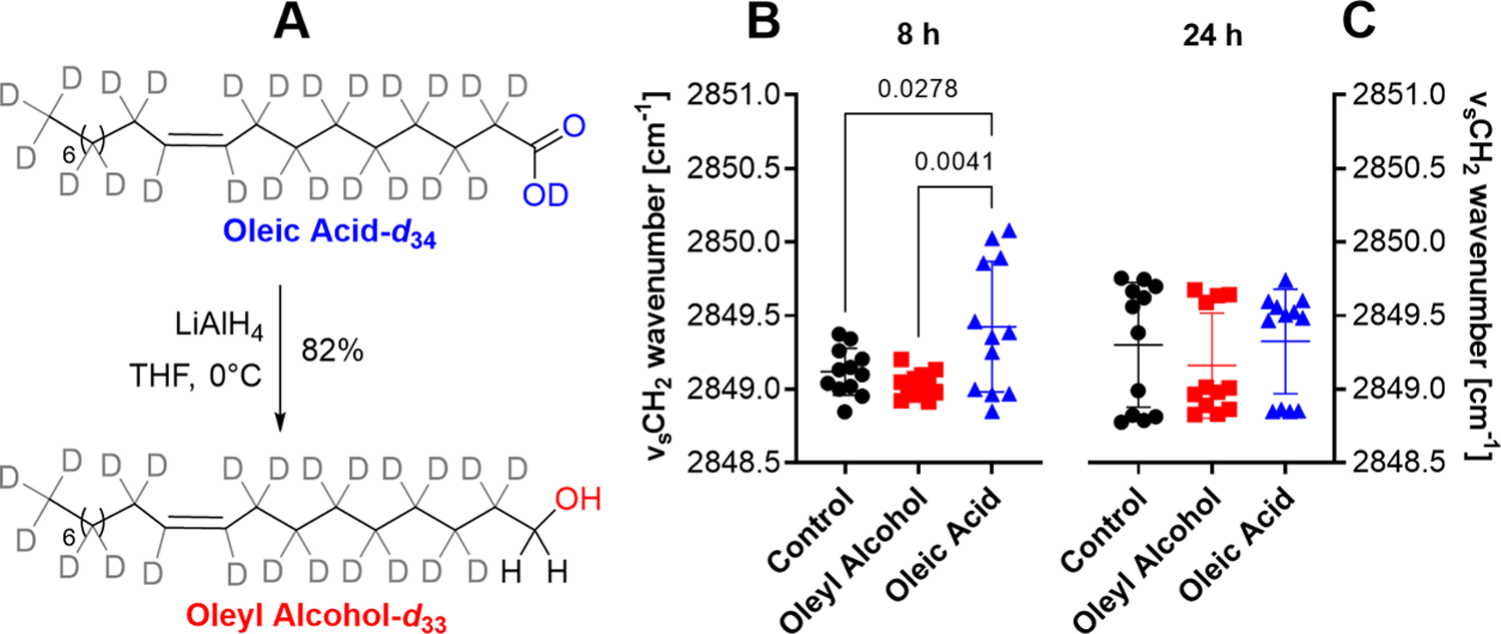
Synthesis of deuterated oleyl alcohol-*d*_33_ (A). Chain order of skin barrier lipids after 8 and 24 h application of oleyl alcohol-*d*_33_, oleic acid-*d*_34_ (0.75% enhancers in isopropyl alcohol/propylene glycol/water 20:6:74, v/v/v at 10 μL/cm^2^), or control (isopropyl alcohol/propylene glycol/water 20:6:74, v/v/v at 10 μL/cm^2^). SC lipid chain order (B) after 8 h and (C) after 24 h application of labeled enhancers. Data are presented as mean ± SD, *n* = 12 (three skin donors, four replicates per donor). The *p* values lower than 0.05 are indicated.

To monitor simultaneously the behavior of the enhancers and the SC lipid chain ordering, we used deuterated enhancers. Oleyl alcohol-*d*_33_ was prepared from oleic acid-*d*_34_ using a LiAlH_4_ reduction (Figure 3A).^35^ Note that the first carbon in oleyl alcohol-*d*_33_ is not deuterated because the hydrogens come from LiAlH_4_; thus, both deuterated enhancers have 33 C–D bonds. We applied 0.75% solutions of oleic acid-*d*_34_ and oleyl alcohol-*d*_33_ to the skin and monitored their effect using FTIR (after washing and drying the skin surface). In the first FTIR experiment, the samples were applied at the dose used in the permeation experiment, i.e., 10 μL/cm^2^ without occlusion. The exposure was terminated after 8 h (i.e., the time at which we observed the permeation-enhancing effect for oleic acid only) and 24 h (i.e., the time at which both enhancers had the same effect on the transdermal permeation of DIC-DEA).

After the 8 h exposure, oleic acid caused a significant red shift in the position of the symmetric methylene stretching vibration compared to both control and oleyl alcohol, indicative of fluidization of the alkyl chains of the SC lipids (Figures 3B and S1A). The same result was found using asymmetric methylene stretching (Supporting Figure S2A). The methylene stretching wavenumbers seen in solvent-treated and oleyl alcohol-treated skin were similar to those before application in this setup. Such lipid fluidization by oleic acid has been reported earlier in superficial SC layers^12^ and model SC lipid monolayers.^10^ Reduced proportion of crystalline lipids was also found in SC lipid models by solid-state ^2^H NMR.^11^ Mak et al. reported fluidized lipid chains in superficial SC layers after 0.5 h exposure to 1% oleic acid in ethanol that propagated to deeper layers within 1.5 h.^14^ In contrast, Ongpipattanakul et al. found that oleic acid increases the conformational freedom of SC lipids only above their transition temperature.^13,36^

**Figure 4:**
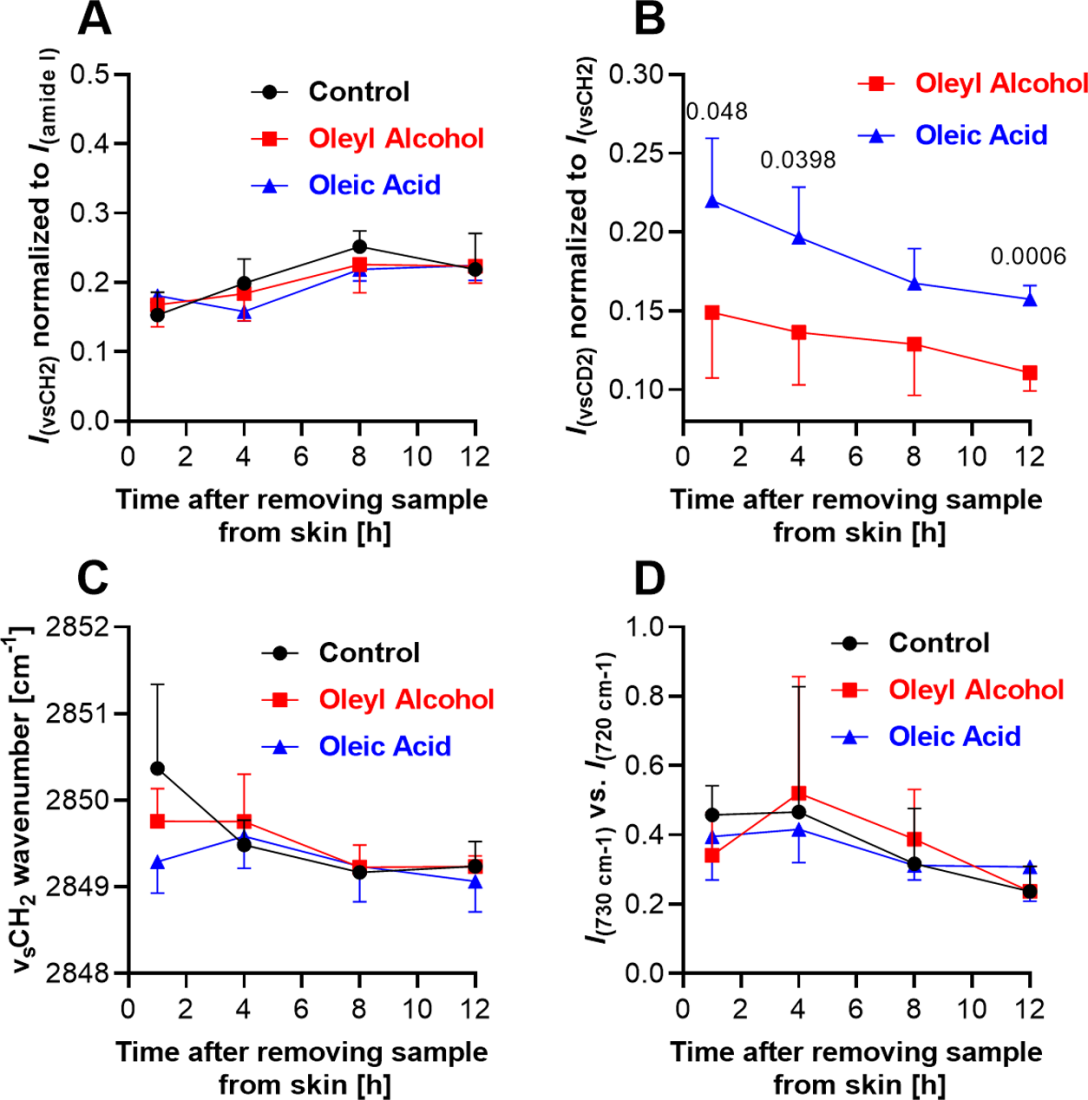
Effects of 24 h treatment with oleyl alcohol-*d*_33_ and oleic acid-*d*_34_ (0.75% enhancers in isopropyl alcohol/propylene glycol/water 20:6:74, v/v/v at 150 μL/cm^2^) on human SC. (A) Relative lipid/protein ratio (shown as the ratio of absolute intensities of methylene symmetric stretching and amide I). (B) Relative enhancer content in the SC lipids (shown as the ratio of intensities of CD_2_ and CH_2_ symmetric stretching). (C) SC lipid chain order (shown as the methylene symmetric stretching position). (D) Relative orthorhombic phase content (shown as the ratio of absolute intensities of the 730 and 720 cm^–1^ rocking vibration). Data are presented as mean ± SD (1 skin donor, *n* = 4). The *p* values less than 0.05 are indicated.

After the 24 h exposure, the position of the methylene stretching vibration was virtually identical for all three treatments (Figures 3C and S1B). The same result was found using asymmetric methylene stretching (Supporting Figure S2B). The ratio of the intensities of the methylene symmetrical stretching vibration and the amide I vibration was also comparable between treatments (same result was found with higher enhancer doses, Figure 4A), indicating that the 24 h application of 10 μL samples does not cause a significant extraction of lipids from the SC. We observed weak vibrations of deuterated enhancers in the CD_2_ stretching region, at wavenumbers consistent with their fluid form (the same result was found with higher enhancer doses, see below and Supporting Figure S3). Thus, it appears that the main mechanism of action of the two enhancers after 24 h of application is likely the formation of fluid domains in the SC lipids. Such phase separation is consistent with previous reports on the action of oleic acid.^11−13^ Due to the very low intensity of these CD_2_ vibrations, we did not attempt to analyze them further. For this purpose, we performed another experiment in which we increased the amount of the sample applied.

### 3.5 Relative Amount of Oleic Acid in the SC Lipids Is Higher Than That of Oleyl Alcohol, Even 12 h after Their Removal from the Skin Surface

To further characterize the interactions of the two enhancers with the SC barrier lipids, we exposed the skin surface to the same amounts of oleyl alcohol, oleic acid, and solvent alone that we used to investigate their effect on the TEWL and electrical impedance (150 μL/cm^2^). The exposure lasted 24 h, after which the solutions were carefully removed, the skin surface was dried with a cotton pad, and FTIR spectra were collected at 1, 4, 8, and 12 h (Figures 4 and S3). Consistent with the above FTIR results, we observed no difference in the relative lipid/protein ratio (Figure 4A), the SC lipid chain conformation (Figures 4C and S4), or the relative content of orthorhombic phase with rigid, tightly packed chains (Figure 4D). A statistically significant difference, however, was observed in the relative content of enhancers in the SC lipids (Figure 4B), measured as the ratio of the intensities of the symmetric stretching vibrations of CD_2_ and CH_2_. The wavenumbers of the CD_2_ vibrations (at approximately 2097 cm^–1^) were virtually identical to those of neat oleic acid and oleyl alcohol, confirming the phase separation of these enhancers and the formation of enhancer-rich fluid domains.

Such disordered enhancer-rich domains may provide an easier permeation pathway for drugs compared to the native SC lipids, either through the domains themselves or through their interfaces with the surrounding lipids. The data in Figure 4B show that the relative proportion of the fluid domains formed by oleic acid is larger than those formed by oleyl alcohol. Figure 4B also shows that the enhancers are slowly washed out of the SC after exposure is discontinued and that the relative concentration of oleic acid in the lipids is higher than that of oleyl alcohol, which could reasonably explain why the effect of oleic acid on electrical impedance, unlike that of oleyl alcohol, is not fully reversible.

## 4 Conclusions

Oleic acid and oleyl alcohol are widely used as skin permeation/penetration enhancers in topical products. Understanding their detailed mechanisms of action and their effects on the skin barrier function therefore is important to drive the choice of one over the other in the early stages of topical product development. Although there are many reports on each of these enhancers in the literature, these data are difficult to compare, as they were collected under different conditions. This study is the first to directly compare the modes of action and reversibility of the effects of oleic acid and oleyl alcohol on the skin barrier function at commercially relevant concentrations. Furthermore, for the first time, we compared directly the two enhancers in their deuterated forms, which allowed us to distinguish between the spectral signatures of the skin lipids and enhancer chains.

This study demonstrated that the actions of oleyl alcohol and oleic acid and their interactions with the human skin barrier differ in several important parameters and that these differences are time-dependent. The skin permeabilization induced by oleic acid is faster than that induced by oleyl alcohol, probably due to the initial fluidization of SC lipids by oleic acid. After 12–24 h, no lipid fluidization is visible, both enhancers form separate fluid domains in skin barrier lipids, and their effect on DIC-DEA permeation is indistinguishable, even though the relative retention of oleyl alcohol in the SC lipids is lower than that of oleic acid. Importantly, the single-dose experiment predicted the result of repeated application (4 doses at 12 h intervals) quite well; that is, both oleyl alcohol and oleic acid increased the permeation of DIC-DEA through the skin about equally (almost 3-fold), and the skin retention of DIC-DEA was lower in the presence of oleic acid than in the presence of oleyl alcohol.

Considering the enhancers, DIC-DEA retention in the skin at 24 h is higher for oleyl alcohol than for oleic acid. The higher content of oleic acid retained in the SC compared to oleyl alcohol persists for at least 12 h after removal of the enhancer from the skin surface, and its adverse effects on the skin barrier integrity indicators (TEWL and electrical resistance) are not fully reversible. This barrier perturbation carries the inherent risk of loss of endogenous body substances, disturbance of epidermal homeostasis, or the permeation of environmental substances and their undesirable interactions in the body.

Thus, oleyl alcohol opens the skin barrier just enough to allow a drug to penetrate through the skin in sufficiently high quantities without a long-lasting negative impact on the skin barrier integrity. Choosing a mild enhancer is important for repeated use of a topical product, especially on fragile or delicate skin.

Although we used DIC-DEA as a representative topical drug, our findings are relevant to other topical drugs that would benefit from facilitating their permeation through the skin barrier. A deeper understanding of the interaction of topical product excipients with the skin and their protective functions is essential to allow their smart choice for future topicals.

## Supporting Information Available

Representative FTIR spectra (CH_2_ symmetric stretching and CD_2_ symmetric and asymmetric stretching bands) and additional FTIR results (comparisons of chain order deduced from asymmetric methylene stretching) (PDF)

## Abbreviations

DIC: diclofenac
DIC-DEA: diclofenac diethylamine
SC: stratum corneum
TEWL: transepidermal water loss
FTIR: Fourier transform infrared
HPLC: high-performance liquid chromatography
PBS: phosphate-buffered saline

## References

[ref1] Williams A. Transdermal and Dermal Drug Delivery: From Theory to Clinical Practice; Pharmaceutical Press: London, 2003 0-85369-489-3.

[ref2] Derry S.; Moore R. A.; Gaskell H.; McIntyre M.; Wiffen P. J. Topical NSAIDs for acute musculoskeletal pain in adults Cochrane Database Syst. Rev. 2015 (6), CD007402 10.1002/14651858.CD007402.pub3. 26068955 PMC6426435

[ref3] Panwar A.; Upadhyay N.; Bairagi M.; Gujar S.; Darwhekar G.; Jain D. Emulgel: A review. Asian J. Pharm. Life Sci. 2011, 2231, 4423.

[ref4] Hagen M.; Baker M. Skin penetration and tissue permeation after topical administration of diclofenac. Curr. Med. Res. Opin. 2017, 33 (9), 1623–1634. 10.1080/03007995.2017.1352497. 28681621

[ref5] Pradal J.; Vallet C. M.; Frappin G.; Bariguian F.; Lombardi M. S. Importance of the formulation in the skin delivery of topical diclofenac: not all topical diclofenac formulations are the same. J. Pain Res. 2019, 1149–1154. 10.2147/JPR.S191300. 31114298 PMC6489664

[ref6] Pradal J.; Vallet C.; Frappin F.; Lombardi M. S.; Cheignon C. Topical delivery of NSAIDs: influence of drug choice, formulation, and dose on in vitro skin permeation. Postgrad Med 2018, 130, 59.

[ref7] Kováčik A.; Kopecna M.; Vavrova K. Permeation enhancers in transdermal drug delivery: benefits and limitations. Expert Opin. Drug Delivery 2020, 17 (2), 145–155. 10.1080/17425247.2020.1713087. 31910342

[ref8] Marjukka Suhonen T.; Bouwstra J. A.; Urtti A. Chemical enhancement of percutaneous absorption in relation to stratum corneum structural alterations. J. Controlled Release 1999, 59 (2), 149–161. 10.1016/s0168-3659(98)00187-4. 10332050

[ref9] Williams A. C.; Barry B. W. Penetration enhancers. Adv. Drug Deliver Rev. 2012, 64, 128–137. 10.1016/j.addr.2012.09.032. 15019749

[ref10] Mao G.; VanWyck D.; Xiao X.; Mack Correa M. C.; Gunn E.; Flach C. R.; Mendelsohn R.; Walters R. M. Oleic acid disorders stratum corneum lipids in Langmuir monolayers. Langmuir 2013, 29 (15), 4857–4865. 10.1021/la4002384. 23517601

[ref11] Rowat A. C.; Kitson N.; Thewalt J. L. Interactions of oleic acid and model stratum corneum membranes as seen by 2H NMR. Int. J. Pharm. 2006, 307 (2), 225–231. 10.1016/j.ijpharm.2005.10.008. 16293379

[ref12] Naik A.; Pechtold L. A. R. M.; Potts R. O.; Guy R. H. Mechanism of oleic acid-induced skin penetration enhancement in vivo in humans. J. Controlled Release 1995, 37 (3), 299–306. 10.1016/0168-3659(95)00088-7.

[ref13] Ongpipattanakul B.; Burnette R. R.; Potts R. O.; Francoeur M. L. Evidence that oleic acid exists in a separate phase within stratum corneum lipids. Pharm. Res. 1991, 8 (3), 350–354. 10.1023/a:1015845632280. 2052523

[ref14] Mak V. H.; Potts R. O.; Guy R. H. Oleic acid concentration and effect in human stratum corneum: non-invasive determination by attenuated total reflectance infrared spectroscopy in vivo. J. Controlled Release 1990, 12 (1), 67–75. 10.1016/0168-3659(90)90184-U.

[ref15] Francoeur M. L.; Golden G. M.; Potts R. O. Oleic acid: its effects on stratum corneum in relation to (trans)dermal drug delivery. Pharm. Res. 1990, 7 (6), 621–627. 10.1023/a:1015822312426. 2367329

[ref16] Tanojo H.; Boelsma E.; Junginger H. E.; Ponec M.; Bodde H. E. In vivo human skin permeability enhancement by oleic acid: a laser Doppler velocimetry study. J. Controlled Release 1999, 58 (1), 97–104. 10.1016/S0168-3659(98)00144-8. 10021493

[ref17] Andega S.; Kanikkannan N.; Singh M. Comparison of the effect of fatty alcohols on the permeation of melatonin between porcine and human skin. J. Controlled Release 2001, 77 (1–2), 17–25. 10.1016/S0168-3659(01)00439-4. 11689256

[ref18] Dias M.; Naik A.; Guy R. H.; Hadgraft J.; Lane M. E. In vivo infrared spectroscopy studies of alkanol effects on human skin. Eur. J. Pharm. Biopharm 2008, 69 (3), 1171–1175. 10.1016/j.ejpb.2008.02.006. 18406117

[ref19] Kim M. J.; Doh H. J.; Choi M. K.; Chung S. J.; Shim C. K.; Kim D. D.; Kim J. S.; Yong C. S.; Choi H. G. Skin permeation enhancement of diclofenac by fatty acids. Drug Delivery 2008, 15 (6), 373–379. 10.1080/10717540802006898. 18686081

[ref20] Zhang Q.; Saad P.; Mao G.; Walters R. M.; Mack Correa M. C.; Mendelsohn R.; Flach C. R. Infrared Spectroscopic Imaging Tracks Lateral Distribution in Human Stratum Corneum. Pharm. Res. 2014, 31 (10), 2762–2773. 10.1007/s11095-014-1373-8. 24792828

[ref21] Ibrahim S. A.; Li S. K. Chemical enhancer solubility in human stratum corneum lipids and enhancer mechanism of action on stratum corneum lipid domain. Int. J. Pharm. 2010, 383 (1–2), 89–98. 10.1016/j.ijpharm.2009.09.014. 19747970 PMC2920799

[ref22] Boelsma E.; Tanojo H.; Boddé H.; Ponec M. Assessment of the potential irritancy of oleic acid on human skin: evaluation in vitro and in vivo. Toxicol. In Vitro 1996, 10 (6), 729–742. 10.1016/S0887-2333(96)00053-7. 20650257

[ref23] Lashmar U. T.; Hadgraft J.; Thomas N. Topical application of penetration enhancers to the skin of nude mice: a histopathological study. J. Pharm. Pharmacol. 2011, 41 (2), 118–122. 10.1111/j.2042-7158.1989.tb06405.x. 2568419

[ref24] Public Assessment Report DE/H/5493 + 6245 + 6603/001–002/DC for Diclofenac AbZ Schmerzgel Diclox/Diclox forte Diclofenac-ratiopharm 2020 https://mri.cts-mrp.eu/Human/Downloads/DE_H_5493_002_PAR.pdf.

[ref25] Kienzler J. L.; Gold M.; Nollevaux F. Systemic bioavailability of topical diclofenac sodium gel 1% versus oral diclofenac sodium in healthy volunteers. J. Clin. Pharmacol. 2010, 50 (1), 50–61. 10.1177/0091270009336234. 19841157

[ref26] Sioufi A.; Pommier F.; Boschet F.; Godbillon J.; Lavoignat D.; Salliere D. Percutaneous absorption of diclofenac in healthy volunteers after single and repeated topical application of diclofenac Emulgel. Biopharm. Drug Dispos. 1994, 15 (6), 441–449. 10.1002/bdd.2510150602. 7993982

[ref27] Zhang Q.; Flach C. R.; Mendelsohn R.; Page L.; Whitson S.; Boncheva Bettex M. Visualization of Epidermal Reservoir Formation from Topical Diclofenac Gels by Raman Spectroscopy. J. Pain Res. 2020, 13, 1621–1627. 10.2147/JPR.S253069. 32753939 PMC7342390

[ref28] Kopečná M.; Kovacik A.; Novak P.; Boncheva Bettex M.; Vavrova K. Transdermal Permeation and Skin Retention of Diclofenac and Etofenamate/Flufenamic Acid From Over-the-Counter Pain Relief Products. J. Pharm. Sci. 2021, 110 (6), 2517–2523. 10.1016/j.xphs.2021.01.022. 33508308

[ref29] Fasano W. J.; Hinderliter P. M. The Tinsley LCR Databridge Model 6401 and electrical impedance measurements to evaluate skin integrity in vitro. Toxicol. In Vitro 2004, 18 (5), 725–729. 10.1016/j.tiv.2004.01.003. 15251192

[ref30] Kopečná M.; Machacek M.; Prchalova E.; Stepanek P.; Drasar P.; Kotora M.; Vavrova K. Galactosyl Pentadecene Reversibly Enhances Transdermal and Topical Drug Delivery. Pharm. Res. 2017, 34 (10), 2097–2108. 10.1007/s11095-017-2214-3. 28664316

[ref31] Kopečná M.; Macháček M.; Nováčková A.; Paraskevopoulos G.; Roh J.; Vávrová K. Esters of terpene alcohols as highly potent, reversible, and low toxic skin penetration enhancers. Sci. Rep 2019, 9 (1), 14617 10.1038/s41598-019-51226-5. 31601936 PMC6787078

[ref32] Roberts M. S.; Cross S. E. A physiological pharmacokinetic model for solute disposition in tissues below a topical below a topical application site. Pharm. Res. 1999, 16 (9), 1392–1398. 10.1023/A:1018998908655. 10496655

[ref33] Kienzler J. L.; Gold M.; Nollevaux F. Systemic bioavailability of topical diclofenac sodium gel 1% versus oral diclofenac sodium in healthy volunteers. J. Clin. Pharmacol. 2010, 50 (1), 50–61. 10.1177/0091270009336234. 19841157

[ref34] Sioufi A.; Pommier F.; Boschet F.; Godbillon J.; Lavoignat D.; Salliere D. Percutaneous absorption of diclofenac in healthy volunteers after single and repeated topical application of diclofenac Emulgel. Biopharm. Drug Dispos. 1994, 15 (6), 441–449. 10.1002/bdd.2510150602. 7993982

[ref35] Opálka L.; Kovacik A.; Sochorova M.; Roh J.; Kunes J.; Lenco J.; Vavrova K. Scalable Synthesis of Human Ultralong Chain Ceramides. Org. Lett. 2015, 17 (21), 5456–5459. 10.1021/acs.orglett.5b02816. 26479675

[ref36] Boncheva M.; Damien F.; Normand V. Molecular organization of the lipid matrix in intact Stratum corneum using ATR-FTIR spectroscopy. Biochim. Biophys. Acta, Biomembr. 2008, 1778 (5), 1344–1355. 10.1016/j.bbamem.2008.01.022. 18298945

