# Supplementary material for: Time-Dependent Differences in the Effects of Oleic Acid and Oleyl Alcohol on the Human Skin Barrier
Source: Mol Pharm. 2023 Nov 11;20(12):6237–45. doi: 10.1021/acs.molpharmaceut.3c00648 (PMC10698716; doi:10.1021/acs.molpharmaceut.3c00648)
Supplement: Supplementary file 1 — mp3c00648_si_001.pdf [file mp3c00648_si_001.pdf]

Supporting Information

Time-Dependent Differences in the Effects of Oleic Acid and Oleyl Alcohol on the Human Skin Barrier

Andrej Kováčik[a], Monika Kopečná[a], Iva Hrdinová[a], Lukáš Opálka[a], Mila Boncheva Bettex[b], Kateřina Vávrová[a,*]

[a]Skin Barrier Research Group, Charles University, Faculty of Pharmacy in Hradec Králové, Akademika Heyrovského 1203, 50005 Hradec Králové, Czech Republic

[b]GSK Consumer Healthcare SARL, a Haleon group company, Route de l'Etraz 2, Case Postale 1279, Nyon 1260, Switzerland

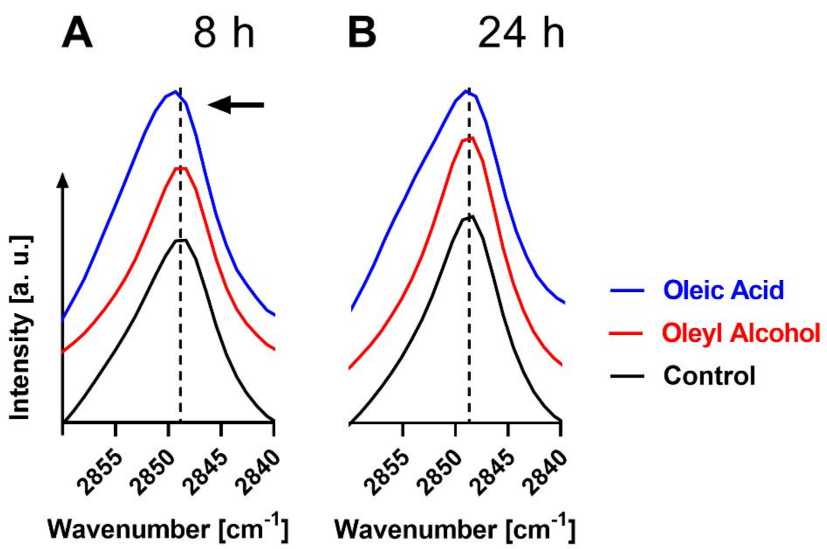

Figure S1. Representative FTIR spectra (methylene symmetric stretching) of skin barrier lipids after 8-h (panel A) and 24-h application (panel B) of oleyl alcohol-d_{33}, oleic acid-d_{34} (0.75% enhancers in isopropyl alcohol/propylene glycol/water 20:6:74, v/v/v at 10 μL/cm^2) or control (isopropyl alcohol/propylene glycol/water 20:6:74, v/v/v at 10 μL/cm^2).

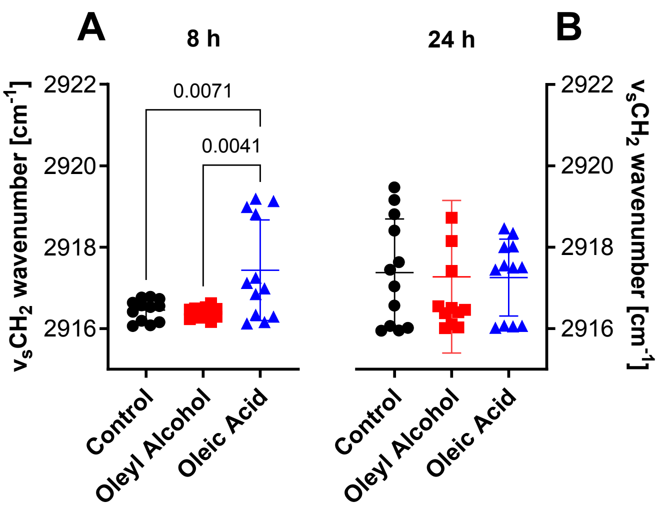

Figure S2. Chain order deduced from asymmetric methylene stretching of skin barrier lipids after 8- and 24-h application of oleyl alcohol-d_{33}, oleic acid-d_{34} (0.75% enhancers in isopropyl alcohol/propylene glycol/water 20:6:74, v/v/v at 10 μL/cm^2) or control (isopropyl alcohol/ propylene glycol/water 20:6:74, v/v/v at 10 μL/cm^2). Data are presented as means ± SD, n = 12 (three skin donors, four replicates per donor). The p values lower than 0.05 are indicated.

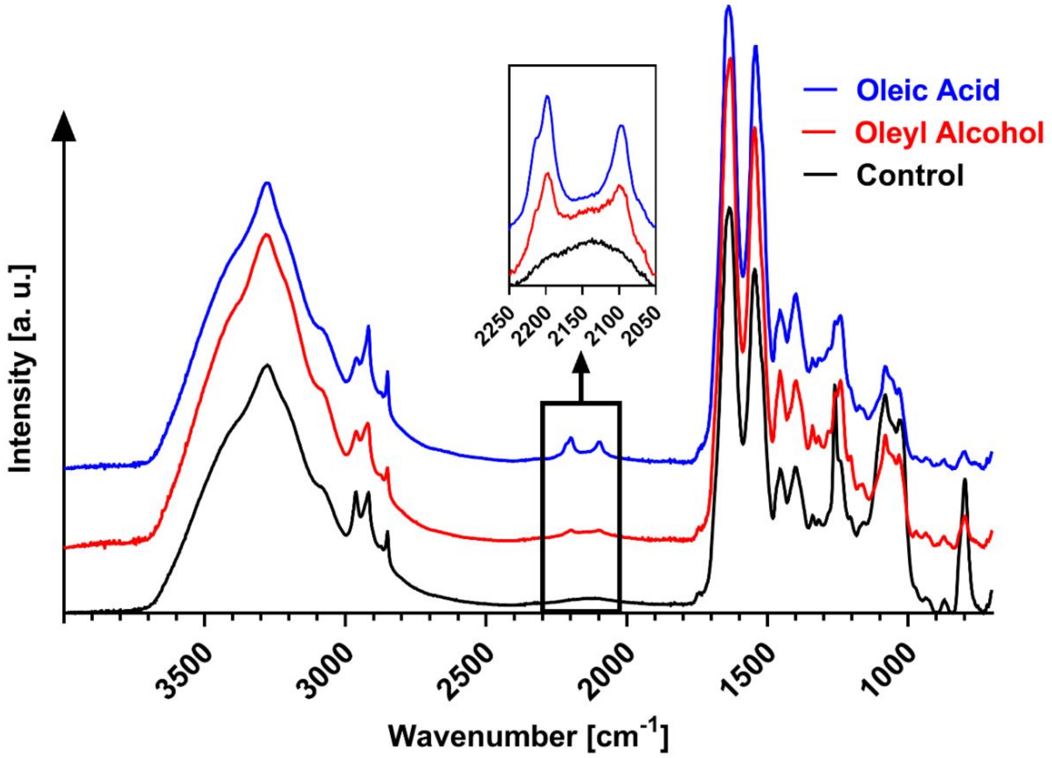

Figure S3. Representative FTIR spectra (region of 4000–600 cm^{-1}) of human skin after 24-h treatment with oleyl alcohol-d_{33}, oleic acid-d_{34} (0.75% enhancers in isopropyl alcohol/propylene glycol/water 20:6:74, v/v/v at 150 µL/cm^2) or control (isopropyl alcohol/propylene glycol/water 20:6:74, v/v/v at 150 µL/cm^2) one hour after sample removal from the skin. The characteristic region for stretching vibrations of deuterated enhancers (2250-2050 cm^{-1}) is given in the insert.

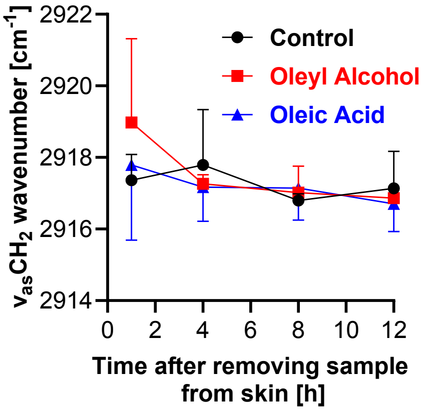

Figure S4. The effects of 24-h treatment with oleyl alcohol-d_{33} and oleic acid-d_{34} (0.75% enhancers in isopropyl alcohol/propylene glycol/water 20:6:74, v/v/v at 150 µL/cm^2) on human SC the methylene symmetric stretching. Data are presented as means ± SD (1 skin donor, n = 4). The p values less than 0.05 are indicated.
